# Novel approach toward the understanding of genetic diversity based on the two types of amino acid repeats in *Erwinia amylovora*

**DOI:** 10.1038/s41598-023-44558-w

**Published:** 2023-10-19

**Authors:** Hyeonheui Ham, Dong Suk Park

**Affiliations:** Crop Protection Division, Department of Agro-Food Safety and Crop Protection, National Institute of Agricultural Sciences, Rural Development Administration, Wanju, 55365 Republic of Korea

**Keywords:** Bacteriology, Genotype, Pathogens

## Abstract

*Erwinia amylovora* is a notorious plant pathogenic bacterium of global concern that has devastated the apple and pear production industry worldwide. Nevertheless, the approaches available currently to understand the genetic diversity of *E. amylovora* remain unsatisfactory because of the lack of a trustworthy index and data covering the globally occurring *E. amylovora* strains; thus, their origin and distribution pattern remains ambiguous. Therefore, there is a growing need for robust approaches for obtaining this information via the comparison of the genomic structure of *Amygdaloideae*-infecting strains to understand their genetic diversity and distribution. Here, the whole-genome sequences of 245 *E. amylovora* strains available from the NCBI database were compared to identify intraspecific genes for use as an improved index for the simple classification of *E. amylovora* strains regarding their distribution. Finally, we discovered two kinds of strain-typing protein-encoding genes, i.e., the SAM-dependent methyltransferase and electron transport complex subunit RsxC. Interestingly, both of these proteins carried an amino acid repeat in these strains: SAM-dependent methyltransferase comprised a single-amino-acid repeat (asparagine), whereas RsxC carried a 40-amino-acid repeat, which was differentially distributed among the strains. These noteworthy findings and approaches may enable the exploration of the genetic diversity of *E. amylovora* from a global perspective.

## Introduction

*Erwinia amylovora* is a plant pathogenic bacterium that causes fire blight disease in apple and pear trees. Historically, this bacterial phytopathogen has led to a significant economic loss in the apple and pear industry worldwide over hundreds of years^1, 2^. Necrosis and blight on blossoms, leaves, and branches are the major symptoms of fire blight, which eventually can induce the death of a whole tree via systemic infection^3^. This destructive microbe is known today as having originated in North America^2^. Currently, *E. amylovora* has quarantine status in many countries outside of North America. Therefore, the outbreak and spread of *E. amylovora* remain a cause for concern, particularly in fire-blight-free countries, such as Australia, Japan, and other countries where apple and pear trees are grown^1^. The first outbreak of *E. amylovora* was reported in the Hudson Valley of New York State in 1793, with subsequent outbreaks occurring in New Zealand, Europe, North Africa, the Middle East, Russia, and Asia^1, 4, 5^. Consequently, the major countries producing and exporting apples and pears have dedicated great efforts to prevent this bacterial pathogen’s invasion or monitor its whereabouts. In turn, this has motivated researchers working on this pathogen, especially phytopathologists, to develop innovative and more powerful approaches for epidemiological investigation and quarantine policy, to investigate, prevent the spread of, or eradicate this microbe in their countries.

Regarding the genotyping of *E. amylovora* strains, many scientists have adopted representative molecular methods to perform epidemiological assays, including clustered regularly interspaced short palindromic repeat (CRISPR)^6–8^, variable number of tandem repeat (VNTR)^9–11^, single-nucleotide polymorphism (SNP) analyses^8, 12–15^, and large chromosomal inversions (LCIs) caused by homologous recombination^16^.

By these methods, *E. amylovora* strains were classified as follows. The *Amygdaloideae*-infecting (AI) group is typically divided into the Widely-Prevalent clade, which comprises isolates from various countries; the Eastern N.A. clade; and the Western N.A. clade. The *Rubus*-infecting (RI) group is genetically distinct from the AI group and exhibits distinct protein profiles. Last, the B-group strains show limited sequence identity to those in either the AI or RI group^13–15, 17^.

However, genotyping markers that can discriminate the *E. amylovora* strains from various countries are highly limited because the genome sequence identity among *E. amylovora* strains is very high, with a homology > 99.5%^18^. As *E. amylovora* has a low genetic diversity compared with other phytopathogenic bacteria, especially in the AI group than the RI group, as well as a chromosome than plasmids^12, 15^, it has been difficult to investigate its genetic diversity, dynamics, and transmission, for AI strains. In addition, SNP analysis based on whole-genome sequencing (WGS) is considered as an advanced genotyping approach with the highly discriminative tool for strain typing in *E. amylovora*^12^. However, this technique is time consuming and costly. In particular, regarding reproducibility, the reference genome and the analytical pipeline and settings should be identical among the various research groups in this field^19^.

Thus, we attempted to perform a comparative genome analysis of the many strains deposited in the public database “GenBank” to improve the molecular markers or approaches generally used for assessing the genetic diversity of *E. amylovora* without the application of WGS techniques and obtain a clearer understanding of the genetic differences among AI strains. Hence, we downloaded and compared all of the *E. amylovora* genome information registered in the NCBI database (http://ncbi.nlm.nih.gov/genome/browse#!/overview) to identify intraspecific genes. Finally, we discovered two types of strain-typing protein-encoding genes, i.e., those encoding “SAM-dependent methyltransferase” and “electron transport complex subunit RsxC”, respectively. The SAM-dependent methyltransferase has a single-amino-acid repeat (asparagine (N)) that is generally more frequent in eukaryotes than in prokaryotes^20, 21^. Remarkably, the number of single-amino-acid repeats in the gene was variable among AI strains but was absent in RI strains. In addition, this gene was located next to the *dnd* operon, which was reported as a genomic island (GI)^18, 22^. Therefore, we also compared the gene composition of AI and RI strains in this region to elucidate the manner in which the difference emerged. The other gene, “*rsxC*”, had tandem repeats composed of 40-amino-acid units toward the C-terminus. The number of repeated amino acids in this gene varied among *E. amylovora* isolates.

This information would help develop new, easy-to-manage genetic markers, thereby reducing time and cost for the strain typing of *E. amylovora*. Furthermore, we believe that the genes discovered in this study will play a crucial role in providing new insights and straightforward answers into the analysis of the genetic diversity of *E. amylovora* with a focus on their distribution pattern and host adaptation.

## Results and discussion

### Amino acid repeat of the “Class I SAM-dependent methyltransferase” protein

**Figure 1:**
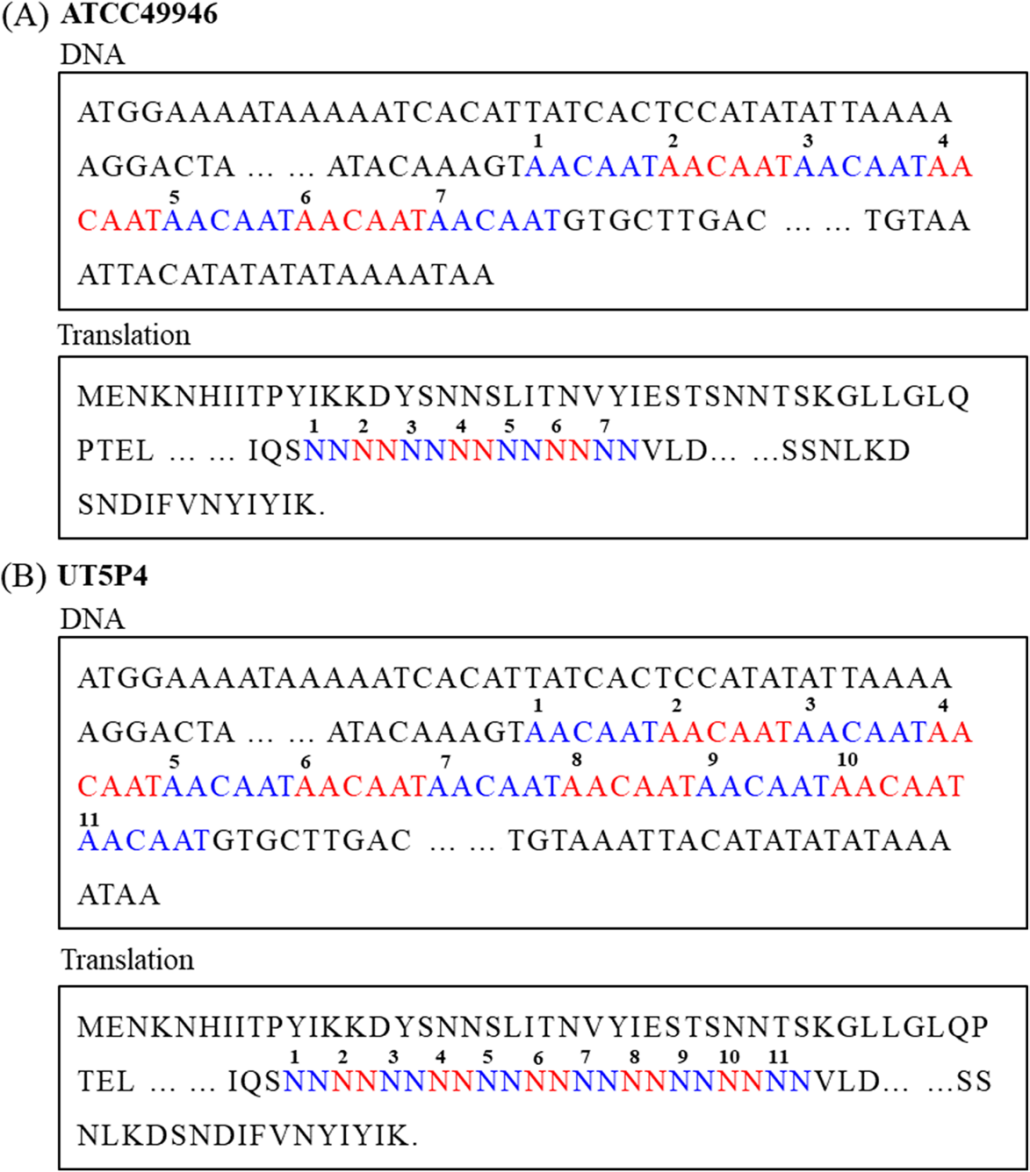
Structure of the hexanucleotide tandem repeats in the gene encoding the ‘Class I SAM-dependent methylfransferase’ and their corresponding single-asparagine repeats. *Erwinia amylovora* ATCC49946 (**a**) and UT5P4 (**b**) strains.

Through a comparative genome analysis of the 245 *E. amylovora* strains downloaded from the NCBI database (Supplementary data-Genome), we found a distinct difference in the size of the gene encoding the “Class I SAM-dependent methyltransferase” (WP_004166224.1 of strain ATCC49946). This gene exhibited sizes ranging from 1326 to 1389 bp (442–463 amino acids) across AI strains. This difference was fully attributable to a hexanucleotide tandem repeat (5′–AACAAT–3′) that ranged from 3 to 15 repeated units (Fig. 1). This repeat encoded two asparagine residues (NN), giving rise to a single-asparagine repeat (SAR) of 6–30 units in the *E. amylovora* strains (Table 1). However, this SAM-dependent methyltransferase gene with a SAR was not detected in most of the RI strains. In addition, we designed PCR primers (metd_F/R) to access SAR from *E. amylovora* and obtained about 405 bp of amplicons from strains 21–18, 21–1, 20–10, and 21–42. After purifying and sequencing the amplicons, we determined their SARs as 18, 20, 22, and 30, respectively.

**Table 1:** Amino acid repeats and basic information of the *Erwinia amylovora* strains.

Strain	Country	Year	Host	SAR number	RsxC Tandem Repeat number	CRISPR type	Group	Accession number
20,070,270	Utah, USA	2007	*Pyrus communis*	6	0		Widely prevalent	GCA_012367975.1
1476	British Columbia, Canada	1997	*Malus domestica*	6	0		Widely prevalent	GCA_012368315.1
Ea6-4	Ontario, Canada	1992	*Malus domestica*	6	0		Widely prevalent	GCA_012371685.1
EaG5	Ontario, Canada	1972	*Pyrus communis*	6	0		Eastern NA	GCA_012367055.1
Ea92-1-2	British Columbia, Canada	2015	*Malus domestica*	12	0		Widely prevalent	GCA_012367095.1
E2005A	Ontario, Canada	1972	*Malus domestica*	18	0		Widely prevalent	GCA_012371795.1
Ea116-5-29	Ontario, Canada	2016	*Malus domestica*	18	0		Widely prevalent	GCA_012367575.1
Ea435	Quebec, Canada	2007	*Malus domestica*	18	0		Widely prevalent	GCA_012367255.1
Ea440	Quebec, Canada	2016	*Pyrus communis*	18	0		Widely prevalent	GCA_012367195.1
Ea169	Israel	N/A	*Pyrus communis*	18	0		Widely prevalent	GCA_012367485.1
1400–1	Washington, USA	1995	*Malus domestica*	20	0		Widely prevalent	GCA_012367905.1
2558	British Columbia, Canada	2008	*Pyrus communis*	20	0		Widely prevalent	GCA_012368075.1
Ea29-7	Ontario, Canada	1992	*Malus domestica*	20	0		Widely prevalent	GCA_012367375.1
O-RG-21	New York, USA	2001	*Malus domestica*	22	0		Widely prevalent	GCA_012366995.1
1668	British Columbia, Canada	1999	*Malus domestica*	22	0		Widely prevalent	GCA_012368135.1
EaD-7	Ontario, Canada	1972	*Malus domestica*	22	0		Widely prevalent	GCA_012371575.1
1617	British Columbia, Canada	1998	*Malus domestica*	6	1		Western NA	GCA_012368155.1
Ea5-97	Nova Scotia, Canada	1997	*Malus domestica*	6	1		Widely prevalent	GCA_012367165.1
Ea6-97	Nova Scotia, Canada	1997	*Malus domestica*	6	1		Widely prevalent	GCA_012367125.1
Ea321	Israel	N/A	*Pyrus communis*	6	1		Widely prevalent	GCA_012367275.1
Ea367	Poland	1996	*Pyracantha* sp.	6	1		Widely prevalent	GCA_012367305.1
Ea650	Poland	1983	*Crataegus monogyna*	6	1		Widely prevalent	GCA_012367155.1
Fb-97b	New Zealand	1993	*Malus domestica*	6	1		Widely prevalent	GCA_012371505.1
Ea12	California, USA	N/A	*Pyrus communis*	12	1		Western NA	GCA_012367545.1
1602	British Columbia, Canada	1998	*Malus domestica*	12	1		Western NA	GCA_012368165.1
245/07	Germany	2007	*Malus domestica*	16	1		Widely prevalent	GCA_012371915.1
214/07	Germany	2007	*Malus domestica*	18	1		Widely prevalent	GCA_012367845.1
IVIA2303	Spain	2000	*Pyrus communis*	18	1		Widely prevalent	GCA_012366985.1
Ea1189	Germany	1989	*Pyrus communis*	18	1	4–24–38	N/A	GCA_016446415.1
1477-1	British Columbia, Canada	1997	*Malus domestica*	20	1		Widely prevalent	GCA_012367885.1
Ea315-1	New Zealand	1994	*Malus domestica*	20	1		Widely prevalent	GCA_012367355.1
Ea1-95	Nova Scotia, Canada	2016	*Rubus idaeus*	–	2		RI clade	GCA_012367475.1
Ea160-3-51	Ontario, Canada	1997	*Pyrus communis*	6	2		Eastern NA	GCA_012371755.1
1482	British Columbia, Canada	2016	*Pyrus communis*	12	2		Western NA	GCA_012368375.1
57671_ID1772_9-Ea_08_07_S49_L001	Italy	2008	*Malus domestica*	6	3			GCA_023184335.1
57679_ID1772_17-Ea_11_13_S57_L001	Italy	2011	*Malus domestica*	6	3			GCA_023184115.1
1279	British Columbia, Canada	1993	*Malus domestica*	6	3		Widely prevalent	GCA_012368325.1
ACW56400	Fribourg, Switzerland	2007	*Pyrus communis*	10	3		Widely prevalent	GCA_000240705.2
ATCC49946	New York, USA	1973	*Malus domestica*	14	3	1–21–38	Widely prevalent	GCA_000027205.1
57663_ID1772_1-Ea_05_07_S41_L001	Italy	2005	*Malus domestica*	14	3			GCA_023184435.1
57669_ID1772_7-Ea_06_13_S47_L001	Italy	2006	*Malus domestica*	14	3			GCA_023184375.1
57670_ID1772_8-Ea_07_08_S48_L001	Italy	2007	*Malus domestica*	14	3			GCA_023184355.1
57675_ID1772_13-Ea_08_34_S53_L001	Italy	2008	*Pyrus communis*	14	3			GCA_023184285.1
57677_ID1772_15-Ea_10_04_S55_L001	Italy	2010	*Pyrus communis*	14	3			GCA_023184225.1
57691_ID1772_29-Ea_13_12_S69_L001	Italy	2013	*Malus domestica*	14	3			GCA_023183905.1
57692_ID1772_30-Ea_14_03_S70_L001	Italy	2014	*Malus domestica*	14	3			GCA_023183945.1
57698_ID1772_36-Ea_15_14_S76_L001	Italy	2015	*Pyrus communis*	14	3			GCA_023183815.1
57699_ID1772_37-Ea_17_01_S77_L001	Italy	2017	*Pyrus communis*	14	3			GCA_023183775.1
57700_ID1772_38-Ea_17_03_S78_L001	Italy	2017	*Malus domestica*	14	3			GCA_023183715.1
57702_ID1772_40-Ea_18_11_S80_L001	Italy	2018	*Malus domestica*	14	3			GCA_023183755.1
57726_ID1772_64-Ea_20_64_S104_L001	Italy	2020	*Cotoneaster* sp.	14	3			GCA_023183225.1
57730_ID1772_68-Ea_20_70_S108_L001	Italy	2020	*Rosa* sp.	14	3			GCA_023183115.1
57731_ID1772_69-Ea_20_71_S109_L001	Italy	2020	*Eriobotrya japonica*	14	3			GCA_023183155.1
57743_ID1772_81-CP_06_S121_L001	Italy	2020	*Cydonia oblonga*	14	3			GCA_023182895.1
21–18	South Korea	2021	*Malus domestica*	16	3		N/A	OQ784852, OR420911
E-2	Belarus	2007	*Malus* sp.	16	3	5–24–38	N/A	GCA_002803865.1
MASHBO	Massachusetts, USA	2015	*Pyrus communis*	18	3	4–27–38	Widely prevalent	GCA_002732135.1
NHSB01-1	New Hampshire, USA	2016	*Malus domestica*	18	3		Widely prevalent	GCA_002732245.1
VTBL01-1	Vermont, USA	2016	*Malus domestica*	18	3		Widely Prevalent	GCA_002732255.1
WSDA87-73	Washington, USA	N/A	*Malus domestica*	18	3		Widely prevalent	GCA_002732215.1
Ea266	Ontario, Canada	1977	*Malus* sp.	18	3		Widely prevalent	GCA_000367565.2
LA635	Cuauhtemoc, Mexico	2014	*Malus domestica*	18	3	5–23–38	Widely Prevalent	GCA_000513415.1
LA637	Guerrero, Mexico	2014	N/A	18	3	5–23–38	Widely prevalent	GCA_000513355.1
01SFR-BO	Ravenna, Italy	1991	*Sorbus* sp.	18	3	4–24–38	Widely prevalent	GCA_000367605.1
UPN527	Navarra, Spain	1996	*Malus* sp.	18	3	4–24–38	Widely prevalent	GCA_000367645.1
NBRC12687	United Kingdom	1959	*Pyrus communis*	18	3		N/A	GCA_000696075.1
Ea356	Germany	1979	*Cotoneaster* sp.	18	3	5–24–38	Widely prevalent	GCA_000367545.2
Ea1/79Sm	Germany	1979	*Malus sylvestris*	18	3	5–24–38	N/A	GCA_015650045.1
CFBP1430	France	1972	*Crataegus* sp.	18	3	4–24–38	Widely prevalent	GCA_000091565.1
CFBP2585	Ireland	1986	*Sorbus* sp.	18	3	4–24–38	Widely prevalent	GCA_000367585.2
57703_ID1772_41-Ea_19_10_S81_L001	Italy	2019	*Pyrus communis*	18	3			GCA_023183725.1
57718_ID1772_56-Ea_20_40_S96_L001	Italy	2020	*Sorbus* sp.	18	3			GCA_023183415.1
57729_ID1772_67-Ea_20_69_S107_L001	Italy	2020	*Pyrus communis*	18	3			GCA_023183195.1
21–1	South Korea	2021	*Malus domestica*	20	3		N/A	OQ784851, OR420910
Ea110	Michigan, USA	1975	*Malus domestica*	20	3	4–23–38	Widely prevalent	GCA_002732505.1
LA636	Cuauhtemoc, Mexico	2014	*Malus domestica*	20	3	5–23–38	Widely prevalent	GCA_000513395.1
57690_ID1772_28-Ea_12_19_S68_L001	Italy	2012	*Malus domestica*	20	3			GCA_023183915.1
57696_ID1772_34-Ea_15_04_S74_L001	Italy	2015	*Crataegus* sp.	20	3			GCA_023183795.1
57697_ID1772_35-Ea_15_08_S75_L001	Italy	2015	*Malus domestica*	20	3			GCA_023183805.1
57701_ID1772_39-Ea_18_05_S79_L001	Italy	2018	*Pyrus communis*	20	3			GCA_023183675.1
57717_ID1772_55-Ea_20_34_S95_L001	Italy	2020	*Pyrus communis*	20	3			GCA_023183455.1
57719_ID1772_57-Ea_20_45_S97_L001	Italy	2020	*Cydonia oblonga*	20	3			GCA_023183375.1
57721_ID1772_59-Ea_20_49_S99_L001	Italy	2020	*Crataegus sp.*	20	3			GCA_023183315.1
57736_ID1772_74-Ea_20_86_S114_L001	Italy	2020	*Sorbus sp.*	20	3			GCA_023182975.1
57739_ID1772_77-Ea_20_121_S117_L001	Italy	2020	*Crataegus sp.*	20	3			GCA_023182945.1
20–10	South Korea	2020	*Pyrus pyrifolia*	22	3		N/A	OQ784850, OR420909
UT5P4	Utah, USA	2020	*Malus domestica*	22	3	7–29–38	Widely prevalent	GCA_002732405.1
57744_ID1772_82-CP_07_S122_L001	Italy	2020	*Ribes* sp.	22	3			GCA_023182935.1
TS3238	South Korea	2015	*Pyrus pyrifolia*	24	3	2–22–38	Widely prevalent	GCA_012980825.1
TS3128	South Korea	2015	*Pyrus pyrifolia*	24	3	2–22–38	N/A	GCA_013375015.1
FB207	South Korea	2015	*Pyrus pyrifolia*	24	3	2–22–38	Widely prevalent	GCA_012980845.1
FB86	South Korea	2015	*Malus domestica*	24	3	2–22–38	Widely prevalent	GCA_012980785.1
FB20	South Korea	2015	*Pyrus pyrifolia*	24	3	2–22–38	Widely prevalent	GCA_012980765.1
17–2187	South Korea	2020	*Pyrus pyrifolia*	24	3	2–22–38	N/A	GCA_017161545.1
CP201324	South Korea	2020	*Malus domestica*	24	3	2–22–38	N/A	GCA_023612655.1
CP200930	South Korea	2020	*Malus domestica*	24	3	2–22–38	N/A	GCA_023612675.1
CP201142	South Korea	2020	*Malus domestica*	24	3	2–22–38	N/A	GCA_023612695.1
CP20140001	South Korea	2020	*Malus domestica*	24	3	2–22–38	N/A	GCA_023612715.1
CP20130204	South Korea	2020	*Pyrus pyrifolia*	24	3	2–22–38	N/A	GCA_023612735.1
CP20086202	South Korea	2020	*Pyrus pyrifolia*	24	3	2–22–38	N/A	GCA_023612755.1
CP20130202	South Korea	2020	*Malus domestica*	24	3	2–22–38	N/A	GCA_023612775.1
CP20161301	South Korea	2020	*Pyrus pyrifolia*	24	3	2–22–38	N/A	GCA_023612795.1
FB307	South Korea	2015	*Malus domestica*	26	3	2–22–38	Widely prevalent	GCA_012980805.1
21–42	South Korea	2021	*Malus domestica*	30	3		N/A	OQ784853, OR420912
CTMF03-1	Connecticut, USA	2016	*Pyrus communis*	10	4		Eastern NA	GCA_002732315.1
CTST01-1	Connecticut, USA	2016	*Malus domestica*	10	4		Eastern NA	GCA_002732295.1
CTBT1-1	Connecticut, USA	2015	*Pyrus communis*	10	4		Eastern NA	GCA_002732385.1
CTBT3-1	Connecticut, USA	2015	*Pyrus communis*	10	4		Eastern NA	GCA_002732205.1
MANB02-1	Massachusetts, USA	2016	*Malus domestica*	10	4		Eastern NA	GCA_002732485.1
MAGFLF-2	Massachusetts, USA	2015	*Malus domestica*	10	4		Eastern NA	GCA_002732175.1
NHWL02-2	New Hampshire, USA	2016	*Malus domestica*	10	4		Eastern NA	GCA_002732435.1
RISTBO01-2	Rhode Island, USA	2015	*Malus domestica*	10	4		Eastern NA	GCA_002732365.1
VTDMSF02	Vermont, USA	2015	*Malus domestica*	10	4		Eastern NA	GCA_002732125.1
MLI181-18	Ohio, USA	2018	*Malus domestica*	10	4		N/A	GCA_019967065.1
MLI200-18	Ohio, USA	2018	*Malus domestica*	10	4		N/A	GCA_019967055.1
LA092	Washington, USA	1988	*Pyrus communis*	12	4	15–34–38	Western NA	GCA_002732285.1
1–2	California, USA	2019	*Malus domestica*	12	4	12–34–38	N/A	GCA_020882215.1
7–3	California, USA	2019	*Malus domestica*	12	4	12–34–38	N/A	GCA_020544325.1
11–7	California, USA	2019	*Malus domestica*	12	4	12–34–38	N/A	GCA_020546585.1
32–10	California, USA	2019	*Malus domestica*	12	4	12–34–38	N/A	GCA_020546605.1
CA3R	California, USA	1995	*Malus domestica*	12	4	8–32–38	B-Group	GCA_002732335.1
OR6	Oregon, USA	N/A	*Pyrus communis*	12	4	14–34–38	Western NA	GCA_002732425.1
OR1	Oregon, USA	N/A	*Pyrus communis*	14	4	12–34–38	Western NA	GCA_002732445.1
HKN06P1	Pennsylvania, USA	2006	*Malus domestica*	22	5		N/A	GCA_004023365.1

We grouped the *E. amylovora* strains according to the SARs number, and then each SAR group’s origin and clade type were analyzed (Table 1). The strains belonging to the Widely-Prevalent clade appeared in various numbers of SAR from 6 to 24. *E. amylovora* strains from various countries, except for some isolates from USA and Canada, belonged to this clade. In Western N.A. clade, SAR 6, 12, and 14 isolated from USA and Canada were included. In Eastern N.A. clade, there were SAR 6, 10, and B-group, SAR 6, 8, and 12 were included. Interestingly, strains of SARs of more than 16 belonged to the Widely-Prevalent clade.

The results of typing *E. amylovora* for the SAR revealed unique patterns in some strains isolated from Korea (more than 24 SAR) but not enough to provide high resolution for typing when used alone. Nevertheless, SAR has only one repeat unit, indicating a comparatively high diversity among *E. amylovora* strains. Thus this repeat region should usually use in combination with other tandem repeat regions as VNTR analysis. Unfortunately, it was difficult to determine the relationship between the host, isolated region, and year according to the SAR length. In addition, strains isolated from *Rubus* spp. did not carry the SAM-dependent methyltransferase gene and SARs, with the exception of the ATCC BAA-2158 strain. This strain, which belongs to the B-group, carried 6 SARs that may be sorted in the AI group, similar to that reported by another study^13, 18^. However, it should be noted that only draft genomes were available for the RI strains.

Generally, bacteria undergo extensive genetic variation in response to various environmental conditions, in part resulting in the expansion and contraction of tandem repeats^23, 24^. In turn, tandem repeats have been reported to undergo insertion or deletion events through slipped-strand mispairing or via uneven cross-over during DNA replication. Therefore, many of the tandem repeat sequences in bacterial genomes have been identified and used as genotyping tools. In the case of *E. amylovora*, tandem repeats have been broadly used in VNTR analysis^9–11^.

In fact, the tandem repeat detected in the gene encoding SAM-dependent methyltransferase was used in a VNTR analysis in another study^9^. However, the repeat was reported as “TAACAA” motif from the target region of the ‘hypothetical protein (CFBP 1430, Eamy_0389)’. Currently, the gene annotation of Eamy_0389 has been changed to “Class I SAM-dependent methyltransferase”, and we revised the repeat motif as “AACAAT”, causing a SAR.

Tandem repeats consisting of multiples of three nucleotides in the coding region generate single-amino-acid repeats in the translated protein^20, 25, 26^. The most-frequently occurring single-amino-acid repeats are glutamine (Q), followed by asparagine (N) and serine (S)^24^. Single-amino-acid repeats have previously been shown to alter protein function or virulence potential^20, 21, 25–28^. Such tandem repeats also happened to cause a SAR in the SAM-dependent methyltransferase gene from AI-type *E. amylovora* strains. However, the functional role of the tandem repeats and the consequences of their variation among strains remain unclear.

### Comparison of the *dnd* and *dpt* operons from *Erwinia amylovora* and *Escherichia coli*

**Figure 2:**
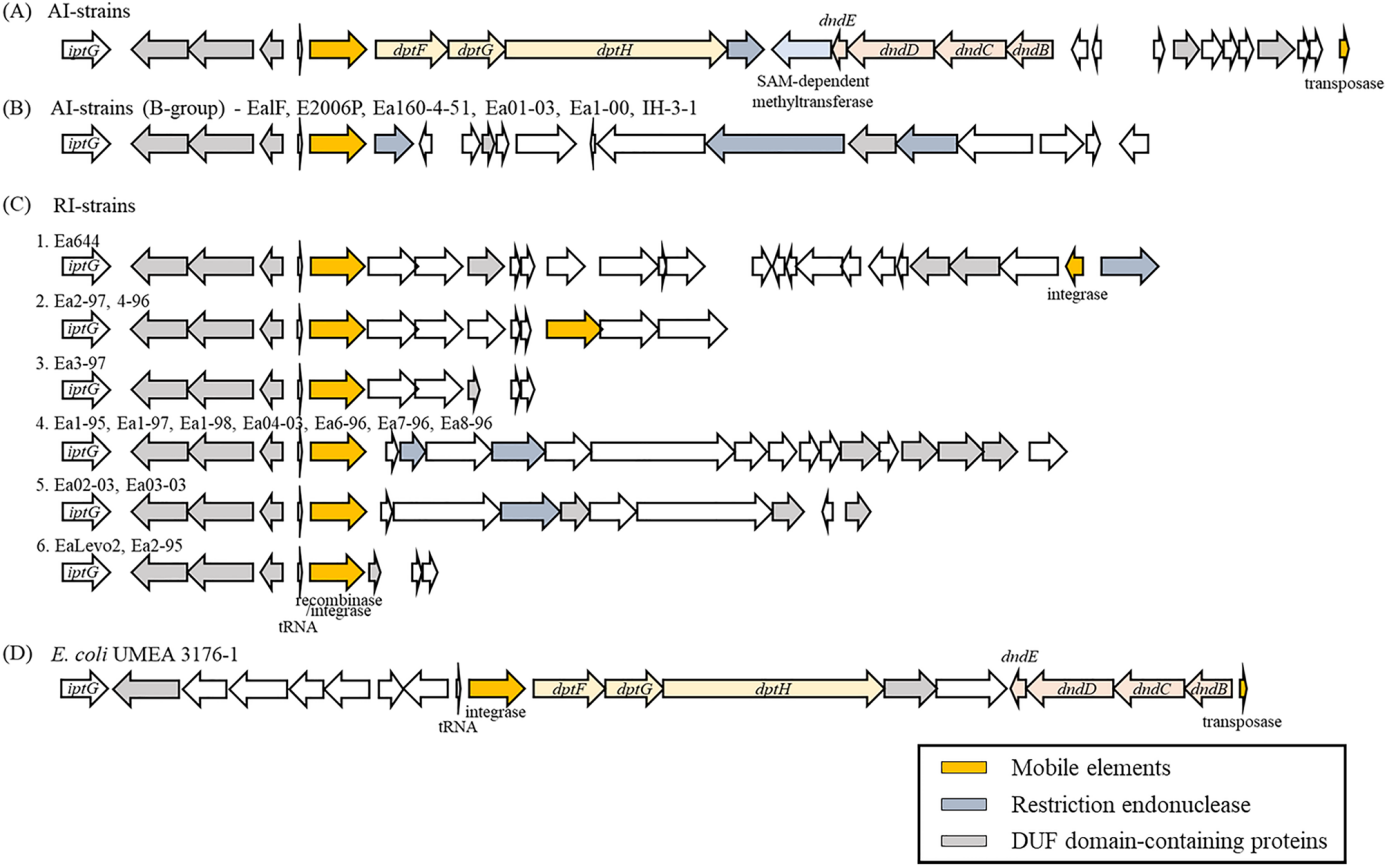
Genetic map of the genomic islands encoding the *dnd* and *dpt* clusters among the different *Erwinia amylovora* strains. Mobile elements (recombinase, integrase, and transposase) are colored in yellow; restriction endonuclease, blue; and DUF domain-containing protein, grey.

We compared the genes surrounding the gene encoding SAM-dependent methyltransferase of AI and RI strains of *E. amylovora* with that of *Escherichia coli* to identify the presence or absence of this gene between the strains (Supplementary data-GI gene components). We detected differences in the gene composition among AI, RI, and B-group strains. In the case of AI strains, a *dpt* gene cluster was observed with *dptFGH* located upstream of the SAM-dependent methyltransferase gene, and a *dnd* gene cluster was detected with *dndEDCB* situated in the downstream region (Fig. 2A). These *dpt* and *dnd* gene clusters were also discovered in the UMEA 3176-1 strain from *E. coli* (GCA_000460595.1), as a similar gene structure. However, genes encoding a hypothetical protein or ATPase instead of SAM-dependent methyltransferase were discovered in *E. coli*^22^. Furthermore, the AI and the *E. coli* strains commonly carried a tRNA and integrase/recombinase gene upstream of *dptF*, which is known as a mobile gene element^22, 29^ and was reported as a GI in the *E. amylovora* CFBP 1430 and ATCC BAA-2158 strains^18^, suggesting that this region was acquired by horizontal gene transfer (HGT). Interestingly, RI strains or some of the AI clades that did not possess the SAM-dependent methyltransferase gene also had both the tRNA and integrase/recombinase genes in this region. However, other genes were present instead of the *dpt*/SAM-dependent methyltransferase/*dnd* cluster. Therefore, some AI strains that did not possess SAM-dependent methyltransferase belonged to the B-group, which carried a specific gene composition after the tRNA and integrase/recombinase gene (Fig. 2B). In addition, RI strains were also clustered differentially according to the gene composition downstream of the tRNA and integrase/recombinase gene (Fig. 2C). Accordingly, we suggest grouping the types of gene structures representing AI, AI B-group, and RI strains in the region located downstream of the tRNA-Leu mobile element and recombinase/integrase gene. Unfortunately, the genomes of all strains presented in Fig. 2B and C were draft genomes, which hampered the full confirmation of the gene structure.

In *E. coli*, the *dnd* operon has been shown to be a GI, and three conserved genes, i.e., *dptF, dptG*, and *dptH*, are found near the *dnd* operon (Fig. 2D). Furthermore, *E. coli* strains encoding the *dnd* operon are frequently among the pathogenic *E. coli*^22^. In *E. amylovora*, RI strains, which are restricted to *Rubus* spp. regarding their host range^30^, did not possess *dnd*/SAM-dependent methyltransferase gene/*dpt* gene clusters in their genome. These observations led us to hypothesise that one of the key factors for determining the pathogenicity and host tropism of *E. amylovora* is the presence of the GI-possessing *dnd* operon. The causal agent of black shoot blight, *E. pyrifoliae*, which has a host range that is limited to specific cultivars of pears and apples and is less virulent than *E. amylovora*^31^, is genetically close to *E. amylovora*, but does not encode this GI. As an extension, studying the host range or pathogenicity of the strains of the B-group, which belongs to the AI strain group, would be valuable for understanding the relationship between the GI and the *dnd* cluster, pathogenicity, and host selectivity after horizontal acquisition.

Since the genes from the EAMY0383-0403 locus of strain CFBP 1430 were determined as a GI^18^, we analyzed the sequence similarity of the gene components in the GI with those of other organisms to explore the origin of GI. As a result, these genes exhibited a high sequence identity with those of *Serratia marcescens* WVU-005, *Klebsiella grimontii* NCTC9146, *Klebsiella pneumonia* RGT40-1, *Yersinia ruckeri* NVI-11050 and YRB, *Yersinia pseudotuberculosis* EP2/ + , *Buttiauxella* sp. WJP83, *Dickeya dadantii* S3-1, *Salmonella enterica* GX1006, *Pectobacterium odoriferum* JK2.1, *Yersinia intermedia* FDDAARGOS_358, *Y. pseudotuberculosis* FDAAGOS_580, and *Pantoea dispersa* Lsch, with a sequence identity of more than 76% and an E-value less than 0.05 (Table 2). Some species were plant pathogens, including *D. dadantii* (for SAM-dependent methyltransferase) and *P. odoriferum* (for *dptG*). However, most of the bacteria were pathogenic to humans and were distributed in soil, water, and the human gastrointestinal tract^32–36^. The taxonomic order of these bacteria was identical, i.e., Enterobacterales.

**Table 2:** Second-order match homology analysis of query genes in the genomic island using the BLASTn module for *Erwinia amylovora* ATCC49946.

Gene	Species	Percent identity (%)	Query coverage (%)	E-value
Transposase	*Serratia marcescens* WVU-005	80.71	98	3e-42
Type II toxin-antitoxin system RelE/ParE family protein	*Klebsiella grimontii* NCTC9146 *substr.* Serovar capsular type 26	97.83	100	2e−128
Ribbon-helix-helix domain-containing protein	*Klebsiella grimontii* NCTC9146 *substr.* Serovar capsular type 26	97.59	100	8e−114
DUF4942 domain-containing protein	*Klebsiella pneumonia* RGT40-1	94	100	0
TA system toxin CbtA family protein	*Klebsiella grimontii* NCTC9146 *substr.* Serovar capsular type 26	96.52	98	5e−143
Type IV toxin-antitoxin system YeeU family antitoxin	*Yersinia ruckeri* NVI-11050	97.49	98	6e−149
DNA repair protein RadC	*Klebsiella grimontii* NCTC9146 *substr.* Serovar capsular type 26	98.94	100	0
DUF932 domain-containing protein	*Yersinia pseudotuberculosis* EP2/ +	95.76	100%	6e−158
Hypothetical protein	*Yersinia rucker* YRB	96.71	99	9e−107
AlpA family phage regulatory protein	*Yersinia rucker* YRB	81.12	98	8e−43
Hypothetical protein	*Yersinia rucker* YRB	97.14	100	0
*dndB*	*Yersinia rucker* YRB	99.8	100	0
*dndC*	*Yersinia rucker* YRB	87.52	100	0
*dndD*	*Klebsiellea pneumonia* INF058-sc-2279968	81.7	99.0	0
*dndE*	*Buttiauxella* sp. WJP83	83.02	89	6e−86
Class I SAM-dependent methyltransferase	*Dickeya dadantii* S3-1	76.10	95	0
Restriction endonuclease	*Salmonella enterica* GX1006	79.17	99	0
*dptH*	*Yersinia intermedia* FDDAARGOS_358	86.98	99	0
*dptG*	*Pectobacterium odoriferum* JK2.1	87.76	100	0
*dptF*	*Yersinia intermedia* FDDAARGOS_358	92.42	100	0
Integrase arm-type DNA-binding domain-containing protein	*Pantoea dispersa* Lsch	90.36	97	0

These results suggest that GI may be horizontally transferred from the *Enterobacteriaceae* pathogens to *E. amylovora*. In a previous study of the genealogy of *Erwinia* spp., *E. amylovora* was shown to have diverged from the enterobacterial ancestor, followed by ancestral *Erwinia*^37^. During evolution, some auxiliary genes acquired by HGT and conferring advantages to certain environmental conditions may have been involved in the evolution and adaptation of bacteria^38^. We also investigated the existence of GI in other bacterial species including *Erwinia tasmaniensis* Et199 (GCF_000026185.1), *E. billingiae* Eb661 (GCF_00196615.1), *E. pyrifoliae* Epk1/15 (GCF_002952315.1), *E. persicina* Cp2 (GCF_019844095.1), *E. rhapontici* BY21311 (GCF_020683125.1), *Pantoea vagans* LMG 24199 (GCF_004792415.1), *P. agglomerans* FDAARGOS 1447 (GCF_019048385.1), *P. ananatis* JBR-LB3-16 (GCF_023611845.1), *Dickeya chrysanthemi* Ech1591 (GCF_000023565.1), *Pectobacterium atrosepticum* 21A (GCF_000740965.1), *Tatumella citrea* ATCC 39140 (GCF_002163605.1), *Brenneria goodwinii* FRB141 (GCF_002291445.1), *Duffyella gerundensis* AR (GCF_020342335.1), and *Mixta hanseatica* X22927 (GCF_023517775.1). However, GI was not found in those species. Interestingly, this GI does not exist in very closely related species, including *E. pyrifoliae* and *E. tasmaniensis*. Therefore, these data have led us to speculate that human activities related to antimicrobials, xenobiotics, heavy metals, or other compounds have a great potential to contribute to the transfer of these genes to *E. amylovora*, eventually conferring genetic diversity and host selectivity to this pathogen.

### Forty-amino-acid repeat located within the “electron transport complex subunit RsxC” gene

**Figure 3:**
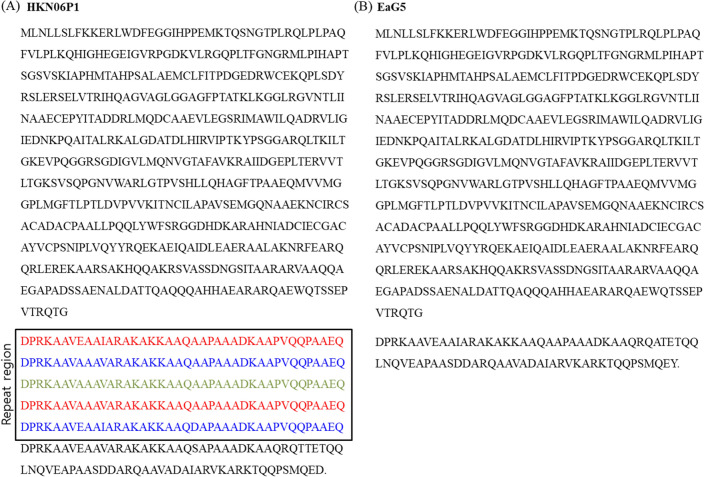
Structure of the 40-amino-acid repeats in the gene encoding the ‘electron transport complex subunit RsxC’ in *Erwinia amylovora.* HKN06P1 (**a**) and EaG5 (**b**) strains.

We found another intraspecific gene, named “electron transport complex subunit RsxC”, with a size that varied among the *E. amylovora* species. The *rsxC* gene was included in the *rsx* cluster in the order of *rsxABCDGE* in *E. amylovora*, and exhibited a similar gene composition to that of the *E. coli rsx* cluster^39^. Among the genes included in the *rsx* cluster, the gene size of *rsxC* alone was different among the *E. amylovora* strains. The size of the *rsxC* gene ranged from 1853 bp (strain EaG5) to 2493 bp (strain HKN06P1), and the main sequence variation among the different *E. amylovora* strains emerged at the position of 1679 bp toward the 3′ end. The translation of the nucleotide sequence of *rsxC* and the comparison of its amino acid sequence between the strains revealed that, starting at amino acid position 553, there were tandem repeats of 40-amino-acid units of the sequence “DPRKAAVEAAIARAKAKKAAQAAPAAADKAAPVQQPAAEQ” toward the C-terminus (Fig. 3). The number of amino acid repeats in *rsxC* varied from 0 to 5 among the *E. amylovora* strains (Table 1). Moreover, we detected this amino acid repeat pattern in both AI and RI strains. Nevertheless, we could not find every amino acid repeat pattern of RsxC in most of the RI strains, because their genome sequence was not complete. In addition, we designed PCR primers (EarsxC_885F/R) for amplifying and detecting amino acid repeats in *E. amylovora*. From the strains 21–18, 21–1, 20–10, and 21–42, 885 bp of amplicons were obtained by PCR and sequenced. Finally, three tandem repeats of 40-amino-acid units were found from each of the strains.

We clustered the *E. amylovora* strains according to the number of amino acid repeats, from rsx-0 to rsx-5, and compared the origin and clade type between the groups. In Widely-Prevalent clade, rsx-0, 1, and 3 which originated from various countries were included. In Western N.A. clade, there were rsx-1, 2, and 4, and in Eastern N.A. clade, rsx-0, 2, and 4 were included. Interestingly, all strains of rsx-3 group belonged to the Widely-Prevalent clade. Unfortunately, the chromosomes of many of the strains that have been deposited in GenBank were in the scaffold or contig form (Supplementary data-genome). From the 16 RI strains deposited in GenBank, we obtained only one *rsxC* sequence from strain Ea1-95, which belonged to the rsx-2 group. Likewise, from the strains of B-group, only strain CA3R had *rsxC* sequence which belonged to the rsx-4 group.

The resolution of this typing method was lower than that of SARs in SAM-dependent methyltransferase since SAR clusters vary from 6 to 30 units. This is because tandem repeats in *rsxC* are composed of 40-amino-acids, and seem to be very conserved and stable. Interestingly, *E. amylovora* strains isolated from North America were classified into each of the amino acid repeat groups. In contrast, the European strains were in the rsx-1 and rsx-3 groups, whereas the Korean strains were only in the rsx-3 group. The genetic diversity of the American strain was higher than that of the European and Korean strains, being proportional to the time of *E. amylovora* emergence. It was also difficult to determine the relationship between the host, isolation region, and year according to the number of amino acid repeats in RsxC.

Intraspecific gene, *rsxC* is also called *rnfC* in other bacteria, and the complex is well known to be related to electron transport using CO_2_ as an electron acceptor in the anaerobic conditions of *Acetobacterium woodii*^39^. The cause of the *rsxC* size difference among the strains is not known; however, the differences in the *rnfC* size among various bacterial species are understood. It has been reported that the RnfC subunit has a FeS center and Flavin- and NADH-binding sites, and that some species have a longer C-terminus^39^. The amino acid repetition causing the size difference in *rsxC* among *E. amylovora* strains was discovered in this study. The exact three-dimensional protein structure of *rsxC* in *E. amylovora* remains unknown. However, repeated units of 40-amino-acid residues may form solenoid or toroid repeats^40^. This sequence repetition trait detected in *rsxC* can be used as a new marker for VNTR analysis.

### Combining and comparing the amino acid tandem repeats with CRISPR spacer patterns

**Figure 4:**
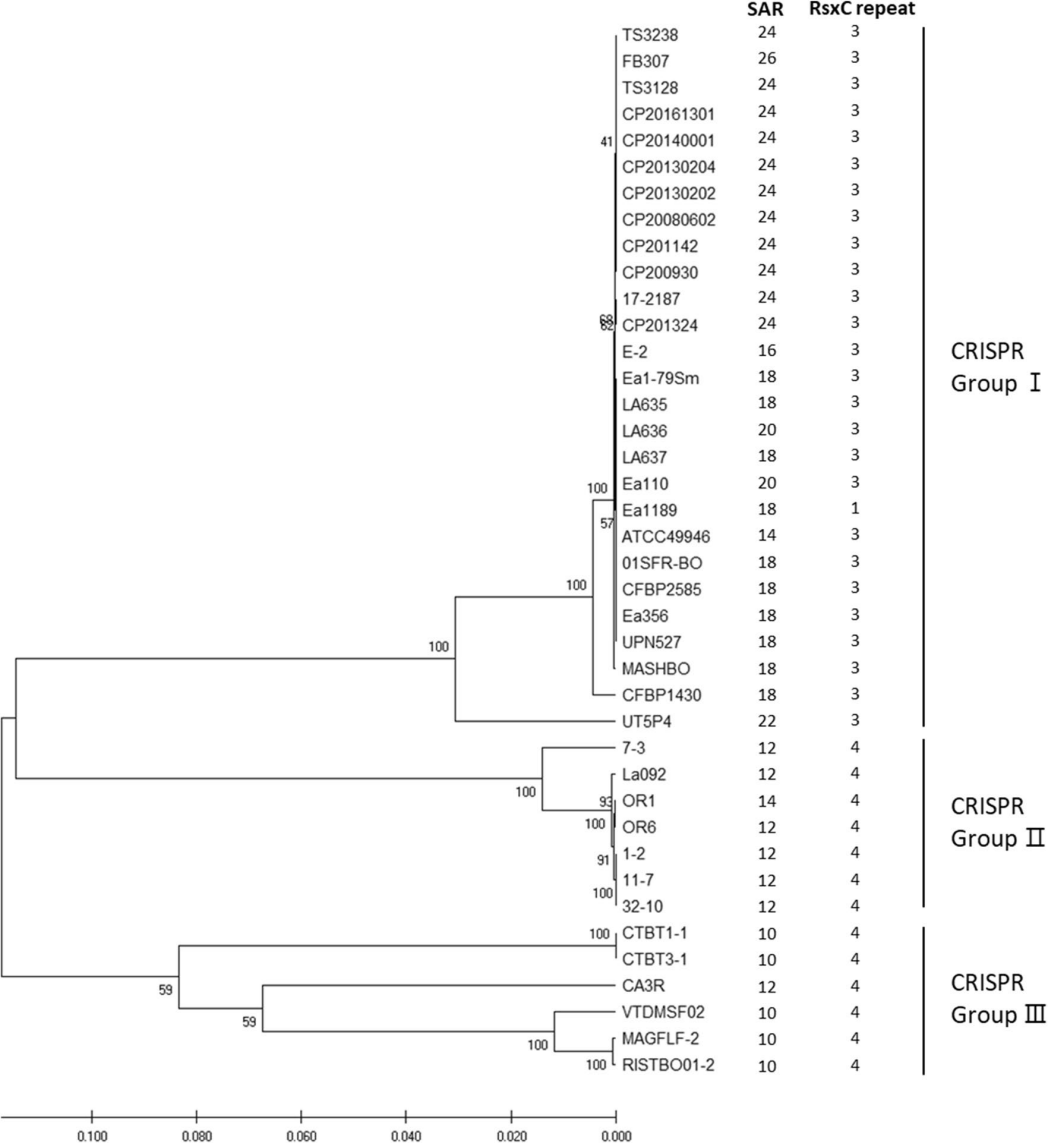
Clustering of CRISPR spacer patterns 1, 2, and 3, and amino acid repeat numbers of *Erwinia amylovora*. SAR; number of single-asparagine repeat in Class I SAM-dependent methyltransferase, RsxC repeat; number of 40-amino-acid tandem repeat unit (DPRKAAVEAAIARAKAKKAAQAAPAAADKAAPVQQPAAEQ) in RsxC.

Additionally, we compared amino acid repeat numbers in SAM-dependent methyltransferase and *rsxC* genes with concatenated CRISPR spacer patterns^6^ (Fig. 4). We could not compare all the *E. amylovora* strains described in this study since a lot of sequences deposited in NCBI appeared as dozens of contigs or scaffolds. However, the clusters made by CRISPR arrays showed regular patterns with amino acid repeat numbers. *E. amylovora* strains were mainly divided into three groups by CRISPR patterns. The strains of CRISPR group I, which were usually belonged to Widely-Prevalent clade was matched with rsx-1, 3 group and 16 to 26 SAR. Whereas most strains of CRISPR group II were belonged to Western N.A. clade, and they were matched with rsx-4 and SAR 12 or 14 group. The strains belonging to CRISPR group III were from Eastern N.A. clade or B-group, and matched with rsx-4 and SAR 10 or 12 group. Suggesting that the resolution of tandem repeats in *rsxC* were more similar to the CRISPR patterns, and SARs would improve the resolution of strain typing by combining these patterns. As LCI types in *E. amylovora* were revealed to describe distribution recently^16^, future studies of combining LCIs with this study would broaden our knowledge about exploring genetic diversity and evolution of *E. amylovora*.

In conclusion, we identified two intraspecific genes, i.e., the “SAM-dependent methyltransferase” and “*rsxC*” genes, using a comparative genomic analysis, to explore the genetic diversity of *E. amylovora*. We found that the differences in the amino acid repeats present in each of these genes detected among the strains caused strain-specific traits and would increase the resolution of epidemiological studies when combined with other typing methods. Furthermore, the SAM-dependent methyltransferase gene, which was flanked by the *dnd* and *dpt* clusters, was only detected in AI strains, and may be acquired by HGT. These results may contribute fundamental information for the study of the genetic diversity and host specificity of *E. amylovora.*

## Materials and methods

### Collection of apple and pear samples

The diseased plant materials collection and use were carried out in accordance with the fire blight surveillance and control guidelines of Rural Development Administration (RDA, Jeonju, South Korea) which is responsible for the management of fire blight diseased orchards. Samples were collected under RDA Phytosanitary Control Officers license (no. 1767). The source of plant samples was listed in the supplementary data-Table S1.

### Bacterial strains and DNA isolation

*E. amylovora* strains were isolated from apple or Asian pear trees with fire blight disease in South Korea. The leaves or branches showing symptoms were sterilised using 70% ethanol, and the margins between the necrotic and healthy tissues were cut into 5 × 5 mm pieces, which were then placed into 1.5-ml microtubes containing 500 µl of sterilized distilled water, followed by grinding and maceration for 30 min. Subsequently, 10 µl of the macerated samples were streaked on tryptic soy agar^41^ and King’s medium B agar^42^, respectively, then incubated at 27 °C for 48 h. Next, a single colony of *E. amylovora* was picked and re-streaked several times to obtain a pure culture. For DNA extraction, *E. amylovora* isolates were cultured in tryptic soy broth at 27 °C and 250 rpm for 24 h, and the cell pellets of the culture were used to extract genomic DNA using a DNA extraction Kit (Wizard^Ⓡ^ Genomic DNA Purification Kit, Promega™, USA), according to the manufacturer’s instructions.

### Whole-genome sequencing

Whole-genome sequencing (WGS) of the *E. amylovora* isolates was performed using the PacBio RSII (Pacific Bioscience, Menlo Park, CA, USA) and HiSeq™ 4000 (Illumina, San Diego, CA, USA) platform combination. Briefly, to construct the library, 8 µg of genomic DNA was sheared to a size of 20–40 kb using a g-TUBE (Covaris, Woburn, MA, USA). Then, using the PacBio DNA template Prep Kit v1.0 (Pacific Bioscience), 10 µL of library was prepared. SMRTbell templates were annealed and sequenced using the DNA/Polymerase Binding Kit P6 and the PacBio DNA Sequencing Kit 4.0 in 8-well SMRT cells, respectively. The subreads were assembled using the Hierarchical Genome Assembly Process v3 protocol and the SMRT Analysis Software v2.3, and the sequences were then corrected and fixed by Quiver v1 and SMRTpipe v2.3.0.139497, respectively. For the HiSeq sequencing, 1 µg of gDNA was randomly fragmented by Covaris, the adapters were ligated at the end of the fragment, and a size of 400–500 bp was selected for PCR amplification. Illumina reads were mapped against the assembled DNA using Pilon v1.21 for sequence compensation.

### Comparative genome analysis

We downloaded the genomic FASTA files of the coding DNA sequences (CDSs) of the *E. amylovora* strains listed in supplementary data (Genome) from the NCBI bacterial genome database (https://www.ncbi.nlm.nih.gov/genome/). We checked the taxonomy and Average Nucleotide Identity results of the deposited sequences in the NCBI Genome Assembly to ensure that the expected sequences were obtained. All collected sequences were compared to mine species-specific genes with more than five differences in amino-acid number in a gene. The nucleotide and amino acid sequences of the mined genes were compared among *E. amylovora* stains using ClustalV of the Lasergene MegAlign software (Version 7.2.1; DNASTAR Inc., Madison, WI, USA). As a result, we discovered amino acid repeats in these genes that varied among the *E. amylovora* strains.

### Primers for analysing amino acid repeats

Two primer sets were designed to directly analyse amino acid tandem repeats from the *E. amylovora* isolates. From both nucleotide sequences of Class I SAM-dependent methyltransferase and *rsxC* genes, forward and reverse primers were designed more than 50 bp outside of each target region. Finally, the metd_F (5′-ATTTATTACGGCTTTGGTTTCTT-3′) and metd_R (5′-CTTTCGATCAGTAGTGTTATTT) primers for detecting SARs in Class I SAM-dependent methyltransferase and EarsxC_885F (5′-GCGGAGTGCGAAACATCA-3′) and EarsxC_885R (5′-GCCTGGCGTGCATCATCTG-3′) for detecting amino acid repeats in *rsxC* were constructed and selected by PrimerSelect software (Version 7.2.1; DNASTAR Inc., Madison, WI, USA). We amplified Korean *E. amylovora* strains 21–18, 21–1, 20–10, and 21–42 listed in Table 1 by metd and EarsxC_885 primers, respectively. The volume of 25 µl reaction mixture was produced by 25 ng gDNA template, 10 mM of each forward and reverse primer, 1 × reaction buffer, 1.25 unit of Taq polymerase (Promega, Madison, WI, USA), and 0.2 mM of dNTPs. The PCR conditions were as follows: pre-denaturation at 95 °C for 5 min, 35 cycles of denaturation at 95 °C for 30 s, annealing at 60 °C (metd) or 69 °C (EarsxC_885) for 30 s, and extension at 72 °C for 40 s, and a final extension at 72 °C for 10 min. The final products were 405 bp (metd) and 885 bp (EarsxC_885) for each primer. The amplicons were purified and sequenced (Bionics™, Daejeon, South Korea) to determine amino acid repeats.

### Structural analysis of the Genomic Island

We compared and analysed the CDS regions located near Class I SAM-dependent methyltransferase in *E. amylovora* strains using BLASTn against standard databases that are publicly available in NCBI genomes (https://blast.ncbi.nlm.nih.gov/). For the BLAST search, we selected “Nucleotide collection (nr/nt) of Standard databases,” excluding organism “*E. amylovora*,” “*E. pyrifoliae*,” and “uncultured/environmental sample sequences,” and program selection optimised for “somewhat similar sequences (blastn).”

### Analysis of CRISPR spacer patterns

To compare amino acid repeat numbers with CRISPR spacer patterns, we collected CRISPR 1, 2, and 3 sequences of *E. amylovora* strains described by McGhee et al. (2012) from NCBI databases. CRISPR sequences were concatenated, aligned, and then clustered by unweighted-pair group method (UPGMA) tree with 1000 bootstrap replications using Mega-X (v 10.0.5).

### Supplementary Information

Supplementary Information.

## Data Availability

The datasets generated and/or analysed during the current study are available in the National Center for Biotechnology Information (NCBI) repository. The sequences related to whole genome sequencing are available at https://www.ncbi.nlm.nih.gov/bioproject/PRJNA734736. Additional information, including the accession number, is presented in Table 1.
